# Technical standardization of ICG near-infrared fluorescence (NIRF) laparoscopic partial nephrectomy for duplex kidney in pediatric patients

**DOI:** 10.1007/s00345-021-03759-6

**Published:** 2021-06-14

**Authors:** Ciro Esposito, Giuseppe Autorino, Vincenzo Coppola, Giorgia Esposito, Mariano Paternoster, Marco Castagnetti, Roberto Cardone, Mariapina Cerulo, Rachele Borgogni, Giuseppe Cortese, Maria Escolino

**Affiliations:** 1 Division of Pediatric Surgery, Federico II University of Naples, Via Pansini 5, 80131 Naples, Italy; 2 Department of Advanced Biomedical Science, Federico II University of Naples, Naples, Italy; 3 Division of Pediatric Urology, University of Padua, Padua, Italy

**Keywords:** Indocyanine green, ICG, Fluorescence, Partial nephrectomy, Children, Laparoscopy

## Abstract

**Purpose:**

This study aimed to standardize the operative technique of indocyanine green (ICG) near-infrared fluorescence (NIRF) laparoscopic partial nephrectomy (LPN) and compare it with the standard technique.

**Methods:**

In the last 4 years, we performed 22 LPN (14 right-sided, 8 left-sided) in children with non-functioning moiety of duplex kidney. Patients included 12 girls and 10 boys with a median age of 3.9 years (range 1–10). Patients were grouped according to the use of ICG-NIRF: G1 included 12 patients operated using ICG-NIRF and G2 included 10 patients receiving the standard technique. We standardized the technique of injection of ICG in three different steps.

**Results:**

The median operative time was significantly lower in G1 [87 min (range 68–110)] compared with G2 [140 min (range 70–220)] (*p* = 0.001). One intra-operative complication occurred in G2. At post-operative ultrasound (US), the residual moiety was normal in all patients. An asymptomatic renal cyst related to the site of surgery was visualized at US in 8/22 (36%), with a significantly higher incidence in G2 (6/10, 60%) compared with G1 (2/12, 16.6%) (*p* = 0.001). Renogram demonstrated no loss of function of residual moiety. No allergic reactions to ICG occurred.

**Conclusion:**

ICG-NIRF LPN is technically easier, quicker, and safer compared with the standard technique. The main advantages of using ICG-NIRF during LPN are the clear identification of normal ureter, vasculature of non-functioning pole, and demarcation line between the avascular and the perfused pole. The main limitation of ICG technology remains the need for specific laparoscopic equipment that is not always available.

## Introduction

Laparoscopic partial nephrectomy (LPN) has become for many pediatric urologists the standard of care to treat pediatric patients with duplex system anomalies associated to a non-functioning moiety [1, 2]. LPN is a difficult procedure to perform [3]. In detail, there are different steps of the operation that are technically challenging due to the complex anatomy of this congenital malformation [4]. At the beginning of the procedure, it is extremely important to safeguard the healthy ureter that is small and difficult to identify because it may be sometimes strictly attached to the dilated ureter of the non-functioning moiety. The dissection of the supplying vessel of the non-functioning moiety is a delicate maneuver and finally it is important to avoid injury of the healthy pole during the resection of the non-functioning moiety [5].

Recently, indocyanine green (ICG) near-infrared fluorescence (NIRF) technology has been adopted in minimally invasive surgery (MIS) with the aim to improve the intra-operative visualization of anatomic structures and the surgical performance during laparoscopic or robotic procedures [6–9].

Indocyanine green (ICG) is a water-soluble fluorescent dye, that can be directly injected intra-venously and it is bounded to albumin or other plasmatic carriers. ICG remains intravascular and allows fluorescent visualization of vessel, lymphatic, and key anatomic landmarks almost instantaneously (< 1 min following the injection) [10, 11].

In the last 5 years, we applied ICG-NIRF technology for several indications including biliary tract and gastrointestinal, oncological, thoracic, and urological diseases [12–15]. In our experience, the main indications of ICG fluorescence technology are in pediatric urology, including laparoscopic partial nephrectomy for benign pathologies such as duplex kidney with one non-functioning moiety [16].

Furthermore, we adopted the ICG-NIRF using the classic ICG camera system and the new RUBINA™ system to perform LPN in patients with duplex kidney anomalies.

This study aimed to standardize the operative technique of ICG-NIRF LPN for duplex kidney in pediatric patients and perform a comparative analysis with the standard technique.

## Materials and methods

In the last 4 years (December 2016–December 2020), 22 LPN (14 right-sided, 8 left-sided) were performed in children with duplex kidney anomalies associated to a non-functioning moiety. Patients included 12 girls and 10 boys with a median age at surgery of 3.9 years (range 1–10). We performed 15/22 (68.1%) upper pole LPN and 7/22 (31.9%) lower pole LPN. The indications for LPN included symptomatic non-functioning upper pole secondary to primary obstructed megaureter (POM) in 12/22 (54.5%) or ureteropelvic junction obstruction (UPJO) in 3/22 (13.6%), symptomatic non-functioning lower pole secondary to UPJO in 2/22 (9.0%) or vesicoureteral reflux (VUR) in 5/22 (22.7%).

The ICG-NIRF was adopted intra-operatively to have a better anatomical definition in the last 12 patients operated on over the last 18 months of the study period. Patients were grouped according to the use of ICG-NIRF: G1 included 12 patients in whom ICG-NIRF was adopted and G2 included 10 patients operated with the standard LPN technique.

The surgical procedures were performed by two senior surgeons, with high volume laparoscopic activity (> 500 laparoscopic procedures per year).

The two groups were compared regarding patient baseline and surgical outcomes, such as operative time, time to full oral feeding, analgesic requirement, length of stay (LOS), intra- and post-operative complication.

Pre-operative work up included renal ultrasound (US) and renal scintigraphy in all patients. In small children (< 12 kg), an intestinal preparation with simethicone, enema, and liquid diet was performed pre-operatively. Follow-up included clinical evaluation and renal US at 1, 6, and 12 months postoperatively and thereafter annually and renal scintigraphy at 12 months postoperatively in all patients.

Statistical analysis was carried out using the Statistical Package for Social Sciences (SPSS Inc., Chicago, Illinois, USA), version 13.0. The demographic data were compared using the Student’s *t* test. The categorical variables were compared using *χ*^2^ test. Significance was defined as *p* < 0.05.

The study received the appropriate Institute Review Board (IRB) approval.

### Operative technique

The parents signed a specifically informed consent before the procedure. Pre-operative antibiotic prophylaxis was administered either with a broad-spectrum medication or according to the child’s specific urine testing.

Patients received a general anesthesia with oro-tracheal intubation and myorelaxation. Before surgery, a cystoscopy was performed to position a catheter into the ureter of the functioning pole. Then, a Foley catheter was positioned into the bladder using sterile precautions and a nasogastric tube was placed to keep empty the stomach during the procedure. The patient was placed in semi-lateral decubitus position. Three trocars were usually placed, but an additional fourth trocar was sometimes needed, more often on the right side, to retract the liver or less frequently on the left side to retract the spleen or the intestinal loops. We preferred 5-mm working trocars to introduce a clip applier for the vessel control or a sealing device and a peanut for the dissection that have 5-mm diameter. The vessels supplying the non-functioning pole were ligated separately with endoscopic clips enabling demarcation of the non-functioning pole. The pole was then excised using special devices. The section line was finally waterproofed by applying a nebulized cyanoacrylate-based synthetic glue with a laparoscopic applicator. The removed moiety was finally extracted through the umbilical orifice. In the patients with associated VUR into the affected kidney moiety, the ureter of the removed moiety was isolated as proximally to the bladder as possible and ligated using Endoloops. At the end of surgery, a drain tube was placed into the abdominal cavity through a trocar orifice and was removed after 24 h.

In the last 12 cases, ICG-NIRF was adopted to have a better anatomical definition during surgery. As for the ICG equipment, a specific camera system and a 30° laparoscope equipped with a special filter for detection of both NIR and standard white light (KARL STORZ SE & Co. KG, Tuttlingen, Germany) were used. A specific software reproduced the NIR image in different colors through selection of different view modes: green or blue (CLARA + CHROMA) or white (SPECTRA A). The specific view mode was selected by the surgeon through the buttons on the camera head during the initial setting. Switching from white light mode to NIRF was directly activated by the surgeon through a foot-pedal.

The last three ICG cases were performed using the new ICG-NIRF RUBINA™ system (KARL STORZ SE & Co. KG, Tuttlingen, Germany). Selecting the overlay mode, the ICG-NIRF data were overlapped onto the standard white light image to generate an overlay image. Depending on your preferences and application, the ICG-NIRF data can be displayed as a green or blue overlay.

Finally, the ICG dye (Verdye, Pulsion Medical Systems, Munich, Germany), available in vials (5 mg/mL), was adopted in all the procedures. The ICG vials were reconstituted with sterile water to create a 2.5 mg/mL solution, which was injected into a peripheral vein or into the ureteral catheter to identify the ureter.

**Fig. 1:**
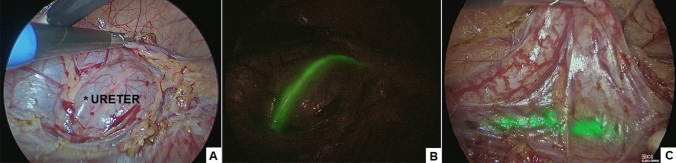
Identification of the normal ureter (*) using white light (**A**), standard ICG-NIRF (**B**), RUBINA™ ICG-NIRF (**C**)

**Fig. 2:**
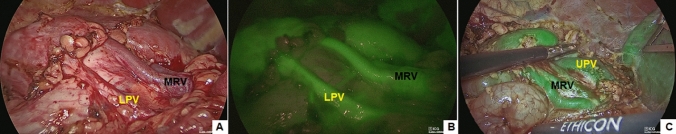
Visualization of main renal vessel (MRV), lower pole vessel (LPV) and upper pole vessel (UPV) using white light (**A**), standard ICG-NIRF (**B**), RUBINA™ ICG-NIRF (**C**)

**Fig. 3:**
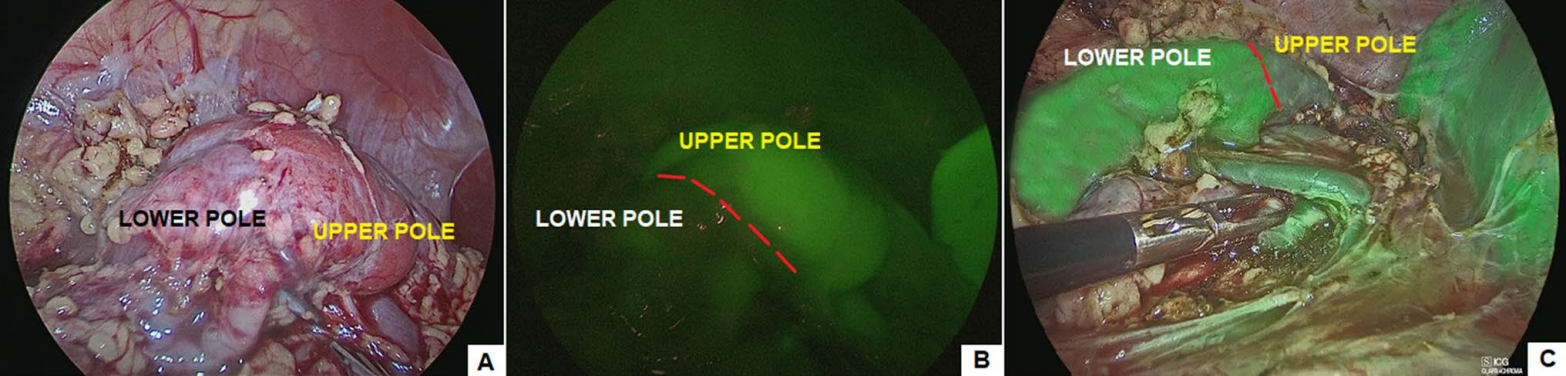
Identification of demarcation line between upper and lower pole using white light (**A**), standard ICG-NIRF (**B**), RUBINA™ ICG-NIRF (**C**)

Target structures appeared fluorescent by 30–60 s following the ICG injection. We standardized the technique of injection of ICG in three different steps. In the first step, a vial of ICG was reconstituted with 10 mL of sterile water and was injected just before surgery into the ureteral catheter to identify the normal ureter (Fig. 1). In the second step, the ICG solution (dosage 0.3 mg/kg/mL) was injected intra-venously to identify the hilar vessel and the vasculature of the non-functioning pole (Fig. 2). In the third step, another bolus of ICG (dosage 0.3 mg/kg/mL) was injected intra-venously, after ligation of the vessel supplying the non-functioning moiety, to identify the boundary plane between the avascular and the perfused pole (Fig. 3) and check the perfusion of the residual pole after parenchymal resection. This third injection ensured that the fluorescent image was especially intense during the parenchymal resection process and remained steady until the end of the procedure, with a mean duration of 55 min (range 35–75).

## Results

Patient baseline, including age at presentation, gender, indications for surgery, were homogeneous in the two groups. All surgical procedures were completed in laparoscopy without need for conversion to open surgery.

The median operative time was significantly lower in G1 [87 min (range 68–110)] compared with G2 [140 min (range 70–220)] (*p* = 0.001). We reported intra-operative complication in one G2 patient, in whom opening of the calyx of the healthy moiety was discovered and repaired intra-operatively using interrupted stitches.

No significant difference emerged between G1 and G2 regarding median LOS, analgesic requirement and time to full oral feeding (*p* = 0.55).

The median follow-up length was 23 months (range 3–48). The residual moiety was normal without parenchymal thinning or hydronephrosis at post-operative US in all patients. However, an asymptomatic renal cyst related to the site of surgery was visualized at US in 8/22 patients (36%). The incidence of these cysts was significantly higher in G2 (6/10, 60%) compared with G1 (2/12, 16.6%) (*p* = 0.001). These cysts disappeared spontaneously, decreased in size or remained unchanged at a long-term follow-up in 7/8 (87.5%) whereas they enlarged in only one case (12.5%) but they did not require any further treatment. Renal scintigraphy demonstrated no post-operative loss of function of the residual moiety in all patients.

No adverse and allergic reactions to ICG and other complications occurred.

Patient baseline and outcomes in G1 and G2 are reported in Table 1.

**Table 1:** Patient baseline and outcomes in G1 and G2

	G1 ICG-NIRF LPN *n* = 12	G2 standard LPN *n* = 10	*p* value
Male/female, n/n	7/5	5/5	0.55
Median age at surgery, years (range)	4.1 (3–10)	3.7 (1–8)	0.33
Side			
Right, *n*	8	6	0.66
Left, *n*	4	4	0.55
Location			
Upper pole, *n*	9	6	0.55
Lower pole, *n*	3	4	0.55
Indications for surgery			
Non-functioning upper pole secondary to POM, *n*	7	5	0.33
Non-functioning upper pole secondary to UPJO, *n*	2	1	0.33
Non-functioning lower pole secondary to UPJO, *n*	1	1	0.33
Non-functioning lower pole secondary to VUR, *n*	2	3	0.33
Median operative time, min (range)	87 (68–110)	140 (70–220)	0.001
Conversion to open surgery, *n* (%)	0	0	n/a
Intra-operative complications, *n* (%)	0	1 (10)	0.33
Median length of stay, days (range)	2.2 (1–4)	2.4 (1–5)	0.55
Median analgesic requirement, h (range)	22 (18–48)	26 (22–54)	0.55
Median time to full oral feeding, h (range)	8 (6–24)	10 (8–24)	0.55
Post-operative complications, *n* (%)	0	0	n/a
Median length of follow-up, months (range)	10 (3–17)	36 (18–48)	0.001
Post-operative renal cyst, *n* (%)	2 (16.6)	6 (60)	0.001
Outcome of renal cyst at follow-up			
Disappeared, *n* (%)	1 (50)	2 (33.3)	0.33
Stable, *n* (%)		3 (50)	n/a
Decreased, *n* (%)	1 (50)		n/a
Enlarged, *n* (%)		1 (16.7)	n/a
Loss of function of residual pole at renal scan, *n* (%)	0	0	n/a

*n/a* not applicable, *POM* primitive obstructed megaureter, *UPJO* ureteropelvic junction obstruction, *VUR* vesicoureteral reflux

## Discussion

Analyzing the adult urology literature, one of the most promising fields of application of ICG-guided fluorescence technology is represented by partial nephrectomy [17–19]. Other applications of ICG-enhanced fluorescence technology were reported in adult robotic urologic surgery and included ureteral reimplantation, radical cystectomy, adrenalectomy and lymphadenectomy after radical prostatectomy [20].

The pediatric literature regarding ICG was mainly focused on ICG application during hepatobiliary surgery to obtain a real-time intra-operative cholangiography and reduce the likelihood of biliary or vascular injuries [13, 21]. In the last 5 years, we extensively adopted this innovative technology in pediatric MIS and standardized the modality of application according to the different indications [22]. In our experience, the main indications of ICG-guided NIRF in the pediatric population included cholecystectomy, lymphatic sparing varicocelectomy, and partial nephrectomy [14].

During laparoscopic partial nephrectomy, ICG technology was first helpful to identify the normal ureter during the dissection of the ureter of the non-functioning pole. Then, thanks to the angiographic properties of ICG, ICG-NIRF provided a real-time intra-operative angiography for identification of hilar vessel and vasculature of the non-functioning pole. After vessel control, ICG-guided NIRF aided to exactly identify the dissection plane between the perfused and the ischemic moiety, thus decreasing the risk of injury to the healthy pole during parenchymal resection or post-operative urinary leakage. Furthermore, it was also useful to check the perfusion of the normal parenchyma following the resection of the affected pole.

We standardized the technique of injection of ICG for partial nephrectomy in three different steps. The first ICG injection was performed just before surgery into the ureteral catheter to identify the normal ureter; the second ICG injection was performed intra-venously to identify the hilar vessel and the vasculature of the non-functioning moiety; the third ICG injection was performed intra-venously, after ligation of the vessel supplying the non-functioning moiety, to identify the boundary plane between the avascular and the perfused pole. This third injection ensured that the fluorescent image was especially intense during the parenchymal resection process and remained steady until the end of the procedure, with a mean duration of 55 min (range 35–75).

We believe that the main advantage of ICG-guided fluorescence technology was the better identification of intra-operative anatomic landmarks, thus allowing more precise dissection and resection and faster surgery. Our series demonstrated that ICG-NIRF LPN was associated with significantly lower median operative time (OT) compared to the standard technique. Considering the shorter follow-up period of the ICG-NIRF LPN group, one could hypothesize that the decreased OT reported in this group may be justified by the improved learning curve and the major experience achieved by the surgeons after the first surgeries performed with the standard LPN. However, the participating surgeons were already experienced with this procedure (> 10 procedures per year) at the beginning of this study. Based upon this data, the evolving learning curve of the participating surgeons should be considered as a confounding factor. In our opinion, the main conditions associated with the significant decrease of OT in the ICG-NIRF LPN were the increased technical facility and the improved anatomic visualization provided by ICG-NIRF imaging.

The better identification of anatomic structures provided by ICG-NIRF also made the procedure safer; in fact, in the group of ICG-NIRF LPN, no intra-operative complication occurred and the incidence of post-operative renal cysts was also significantly lower compared with the standard technique.

The exact etiology of these cysts remains uncertain but it is probable that a seroma takes the place of the removed pole [23]. As these collections can be due to the persistence of some excretive structures of an incompletely resected kidney moiety, parenchymal resection should be performed exactly in correspondence of the demarcation line of the ischemic pole to be removed, that is usually visualized after vessel division [23]. If you cut the ischemic parenchyma above this line, there is a higher risk to leave residual ischemic parenchymal tissue that may predispose to cyst formation.

We already published in 2017 that ultrasonographic diagnosis of an avascular cyst related to the operative site was reported in approximately half of LPN performed over a 10-year period (48.8%) [23]. In the standard LPN group of this study, an even higher incidence (60%) of post-operative cysts was reported, although the LPN had been performed by the same authors in a later period compared with the previous series [23]. Furthermore, the two asymptomatic renal cysts in the ICG-NIRF LPN group were not encountered during the initial experience with this technology. These findings may allow to exclude a direct correlation between formation of post-operative cysts and the laparoscopic partial nephrectomy experience of the surgeons involved in the study. Our results again suggested that the main conditions, that may be associated with the lower incidence of post-operative cysts in the ICG-NIRF LPN compared with the standard LPN, were the excellent intra-operative visualization of the boundary plane between the ischemic and the perfused pole and the more precise parenchymal resection provided by ICG-NIRF imaging.

This technique was no time-consuming since it just required an intravenous injection of ICG solution and fluorescence of target tissue/organ was visualized in real-time intra-operatively. Furthermore, using our standardized technique of ICG administration, the fluorescent image remained steady until the end of the procedure and particularly during the parenchyma resection phase.

In our series, the adoption of ICG fluorescence technology was also cost-effective; in fact, since the operating room was already equipped with the specific ICG-NIRF camera system, use of ICG-NIRF in laparoscopy did not require any additional costs except for the ICG dye (40 eur/vial). Furthermore, ICG was clinically safe, non-nephrotoxic and no allergy and other adverse systemic reactions were reported in the early or late post-operative course. We adhered to the general recommendation that ICG not be used for patients with a shellfish allergy or iodine contrast sensitivity [24, 25].

In our series, two different ICG camera systems were adopted to perform ICG-NIRF LPN. The standard system allows switching from standard view to ICG-NIRF by pushing a foot-pedal and the screen becomes black and white and the target structures appeared fluorescent. The new ICG RUBINA™ system allows switching from standard view to ICG-NIRF by pressing a button on the camera head and, in the overlay mode, the ICG-NIRF data are overlapped onto the standard white light image to generate an overlay image. Thus, using the overlay mode of ICG-NIRF provided by RUBINA™, we could adopt NIRF imaging throughout the entire operation, without the need to switch between standard camera views to those with ICG-NIRF to definitively identify the kidney anatomy.

Based upon our preliminary experience, we believe that the new RUBINA™ technology is preferable to the standard system, due to the possibility to overlay the ICG-NIRF data onto the standard white light image and provide surgeons a constant fluorescence imaging of the target anatomy.

The results of this study confirmed that ICG-NIRF LPN was technically easier, quicker and safer compared with the standard technique. Based upon our experience, the main advantages of using ICG fluorescence technology during LPN include the clear identification of the ureter of the normal moiety, the excellent exposure of hilar vessel and vasculature of the non-functioning pole and the clear identification of the demarcation line between the avascular and the perfused pole. It is mandatory to standardize the technique of injection of ICG to achieve these good results. The main limitation of ICG technology remains the need for the specific laparoscopic equipment that is not always available in all surgical units.
